# Probiotics in Rabbit Farming: Growth Performance, Health Status, and Meat Quality

**DOI:** 10.3390/ani11123388

**Published:** 2021-11-26

**Authors:** Simone Mancini, Gisella Paci

**Affiliations:** Department of Veterinary Sciences, University of Pisa, Viale delle Piagge 2, 56124 Pisa, Italy; gisella.paci@unipi.it

**Keywords:** *Bacillus*, *Lactobacillus*, *Enterococcus*, *Saccharomyces*, GIT, health status

## Abstract

**Simple Summary:**

Probiotics are microorganisms that can interact with the host and with the other microbiota present in the gastrointestinal tract of the host. Rabbits’ digestive characteristics are based on highly specialised colonies of intestinal microorganisms, which makes them vulnerable to metabolic disorders. Probiotics can balance gut microbiota and have several positive effects on the health status of the animal, leading also to the increase of the growth performance and meat quality.

**Abstract:**

The rabbit’s complex microbiota of the gastrointestinal tract (GIT) plays a critical role in feed digestion, in vitamin production, in fermentative activity with production of volatile fatty acids, and stimulation of immune response, as well as in the infection defence against pathogens and countering environmental stresses. To prevent digestive disorders of this fragile ecosystem, rabbit breeders adopt suitable diets supplemented with additives such as probiotics. Probiotics can interact with the host and with the other microflora leading to an increased health status. A review on the effects of probiotics on rabbit growth performance, health status, and meat quality was conducted to reduce the incidence of digestive diseases and enhance productive performance. Some authors observed that the supplementation of probiotics to the diet improved feed conversion ratio and growth and digestion coefficients, while other authors reported a lack of effect on the live performance. Benefits derived from the use of probiotics were observed on the mortality and the morbidity. The studies performed, to evaluate the effects of probiotic supplementation in diets on the immune response, showed variations in the results. Some authors reported no significant effect on haematological parameters, such as total protein, immunoglobulins, and IgG, while others observed a significant increase or decrease of the same parameters. Most of the research reported significant modifications of intestinal morphology and positive effects on the GIT microbiota, supporting the host’s natural defences. Regarding the carcass and meat quality, the studies reported only partial and opposing results.

## 1. Introduction

Rabbit represents one of the most interesting production animals as, theoretically, it is an ideal meat-producing animal. Indeed, rabbit has a short life cycle, it is very prolific, has a short gestation period, and it has a high feed conversion capacity (2–2.3 on high grain diets, and 3–3.8 on high forage, grain-free diets) [1,2]. The rabbit is a monogastric hindgut fermenter, as via caecotrophy its digestive physiology allows it to obtain proteins and vitamins. Despite all these important features, rabbit consumption is decreasing worldwide, mostly in relation to the consumers’ acceptance and the requested cooking time [3]. Thus, rabbit farmers, also in Mediterranean countries in which rabbit meat was popular in the last decades (France, Italy, and Spain), are facing a market severity and a decrease of the meat request [4]. On the other hand, rabbit meat could be “re-discovered” by food producers as a healthy food as it is rich in protein while low in fat, cholesterol, and sodium [5]. It could be also proposed as an alternative to the conventional meat-based products (mostly containing beef and pork), especially for children and the elderly [3]. Furthermore, ready-to-cook products could be well accepted by consumers and meet new market trends [6,7].

Despite this regression in European countries, rabbit farming is becoming an important emerging business in the developing countries, mostly in relation to the abovementioned productivity and to the already established highly specialised farming procedures, the technically advanced and unique livestock industry [8]. Farming stress is very critical in rabbit farming, mostly in hot environments. Rabbit farming faces a very critical step in the weaning period, as kits are separated from the mothers and solid feed replaces maternal milk [9]. During this period, as a consequence of environmental and physiological changes, rabbits are easily stressed and subject to non-specific enteritis and gastrointestinal infections, normally linked to dietary stresses, to parasites (*Coccidia*) and bacteria (*Clostridia* spp. and enteropathogenic *Escherichia coli*), leading to a multifactorial gastrointestinal syndrome (epizootic rabbit enteropathy, ERE) [10].

## 2. Probiotics

The term probiotic derives from two words: pro of the Latin language and βίος of the Greek language and literally means “for life” [11]. The history of probiotics began with the civilisation of humans: since the times of the Greeks and the Romans, the properties of fermented products were known, and their consumption was recommended both in children and in convalescents [12]; they are mentioned also in the Bible and in the sacred books of Hinduism. Particularly, they were identified in cultured dairy products. As reported in some studies, it seems that “fermented dairy products” were known also by Sumerians and that pictorial finds on the treatment of milk emerged during excavation in ancient Mesopotamia [11]. Moreover, archaeologists found evidence of fermented products derived from rice, fruits, honey, and cereals in Neolithic villages in China, Egypt, and Mesopotamia [11]. Moreover, Homer and Plinio tell of fermented milk and suggest its use in the treatment of gastrointestinal infections [11]. At the origin, these products were considered part of “folklore” until the healthy properties began to make their way and they were used as functional food linked to the presence of probiotic bacteria [11]. Considering the literature, probiotic is a relatively new word used to indicate microorganisms being able to provide healthy effects in humans and animals [12].

At the beginning of the 20th century, the role of gut flora was unknown and in 1908 it was Metchnikoff who began to study the role of bacteria involved in fermentation of the intestinal flora and laid the foundation for the development of what we actually name “probiotic microorganism” [12,13]. At the time, many researchers were sceptical about the use and the healthy promoting effect of bacteria on the intestinal tract and opposed the bacterial therapy [12]. It was only in the first decades of 1900 that the therapeutic effects of *Lactobacillus* and *Bifidobacterium* were documented, and researchers began to believe that these microorganisms were essential to keep the digestive tract healthy [12,14,15].

Only at the end of the century the role of gut flora and the protective function of bacteria against pathogens were cleared and some fermented foods were considered probiotic products due to the presence of one or more probiotic bacteria [12].

The first study that proposed the term “Probiotika” was Kollath in 1953 to indicate substances opposite to antibiotics and “connected with vital processes” [13]. Rusch [16], in a short overview, reported the definitions of the term probiotic used by different authors and observed that the term is controversial. In most cases, the authors agree with the definition proposed in 1974 by Parker [17], who described the probiotic as “Organism and substance which contributes to intestinal microbial balance” [13,18]. Subsequently, in 1989, Fuller proposed another definition and considered the probiotic as “a live microbial feed supplement which beneficially affects the host animal by improving its intestinal microbial balance” [19]. The US FDA (Food and Drug Administration) uses the term “direct feed microbial” (DFM) instead of probiotics and suggests the following definition: “DFM is a source of live naturally occurring micro-organism” and included bacteria, fungi, and yeast [13,18].

The EU legislation does not report a definition of probiotics but particularly the Regulation (EC) No 1831/2003 establishes the additives for use in animal nutrition and included the microorganism as “feed additives” and established the conditions for authorisation. Among the conditions, the Regulation reports the capacity of feed additives “to affect favourably animal production, performance or welfare, particularly by affecting the gastro-intestinal flora or digestibility of feeding stuffs”. The same Regulation establishes the conditions to obtain the authorisation for the use of feed additives. The request for authorisation must be sent to the European Commission that forwards the application to the European Food Safety Authority that shall give an opinion regarding the application. EFSA reports that the probiotics are substances that improve the equilibrium of the intestinal tract microflora [20].

Different bacterial strains have different probiotic potential and differences are within the same species. The different strains have specific areas of adherence (site-specific), exact immunological effects, and different modes of actions if in the presence of a healthy or inflamed gastrointestinal tract. The goal of the researchers involved in the studies of probiotic microorganisms was to understand the interactions between the supplemented microorganisms and the microbiota of the host and define possible probiotic bacteriotherapy applications.

However, not all the research studies agreed on the role of probiotics. Despite the numerous definitions, some authors consider the problem of probiotics a question that needs further clarification and in particular suggest the use of strict criteria before considering a substance as probiotic or not [12]. For example, Havenaar et al. [21] established a new concept of probiotics, basing their evaluation on strict criteria such as the resistance to gastric acidity and pancreatic secretions, the adhesion to epithelial cells, the presence of antimicrobial activity, the inhibition of adhesion of pathogenic bacteria, the evaluation of resistance to antibiotics, the tolerance to feed additives, and the stability in the feed matrix.

To describe the mechanism of action of probiotics, non-specific terms are generally used. To indicate biological effects of probiotics, authors report terms such as colonisation resistance or competitive exclusion. However, probiotic microorganisms might be able to control commensal and/or pathogenic ones, neutralising the toxic effect of pathogens, increasing the host’s defences from the immune system, and showing antioxidant activity [12,21,22].

The microbiota, represented by a great number of microbial agents which colonise specific ecological niches, contribute to the health status of the gastrointestinal tract due to its multiple functions.

The animals’ complex microbiota of the gastrointestinal tract (GIT) plays a critical role in feed digestion, in vitamin production, in fermentative activity with production of volatile fatty acids, and in the stimulation of immune response, as well as in the defence against pathogen infection and a hostile environment. Moreover, modifications in villus height and crypt depth, which are considered the major markers of gut development, health, and functionality, can be influenced by gut bacteria.

The control and the balance of rabbit microbiota contribute to produce positive effects on productive performance and health. In rabbits, the alteration of microbiota *equilibrium* can determine pathological effect, such as enteritis producing economic losses in rabbit farms. In general, the aetiopathogenesis of intestinal inflammatory processes is caused by multifactorial factors, such as environmental, nutritional, age, management, and pathogenic agents (*Escherichia coli*, *Clostridium spiroforme*, *Lawsonia intracellularis*, *Clostridium piliforme*, *Salmonella* spp., rotaviruses, coronaviruses, parvoviruses, and astroviruses) [23].

To prevent digestive disorders of this fragile ecosystem, breeders adopt suitable diets supplemented with additives such as probiotics.

In general, probiotic properties are evaluated in vitro by testing their antimicrobial potential, ability to adhere to the host’s intestinal mucin, and resistance to the gastrointestinal environment. Meanwhile, other probiotic properties, expressed in vivo, are more difficult to be evaluated, such as the ability to stimulate the development of the intestinal immune system and the ability to regulate intestinal innate immune and inflammation homeostasis.

Moreover, different mechanisms of action have been ascribed to probiotics, even if some of them are hypothetical. The probiotics contribute to the maintenance of the gut habitat in eubiosis, probably preventing the entry to and the gut colonisation of pathogenic bacteria, increasing the activities of commensal bacteria, producing inactivation of toxins, and detoxification of host and nutrients in the gut.

## 3. Effects of Probiotics on Rabbits

During the last decades, several research studies were published about the effects of probiotics on rabbit farming. As the research articles deal with several different factors, such as rabbit breeds, sex, ages, type of basal diets, type of probiotics administration, duration of the trials, environmental conditions (rearing climate), etc., we report here a review of the main results focusing our attention on the productive performance of rabbits (i.e., studies about the use of rabbits intended as experimental animals were not taken into account) and how probiotics could contribute, due to their capacity to interact with feedstuffs, with other microorganisms, and with their host, to affect rabbits’ health status and production. Experimental designs of the reviewed research articles can be found in Table 1.

### 3.1. Live Performance

Probiotics could have a role in rabbit weight gain and in the capacity of the animals to assimilate the nutritional value of the feedstuffs and positively convert them into body mass. Feed conversion ratio (FCR) could be positively affected by probiotic metabolisms that might contribute to a better use of feeds, as also metabolised probiotic cells could be part of the assimilated nutrition.

Abdel-Wareth et al. [24], who tested supplementation of mixes of fenugreek seeds and probiotics (AmPhi-Bact, American Pharmaceutical Innovations Company^®^, containing a mix of lactic acid bacteria culture, *Lactobacillus acidophilus*, *Lactobacillus plantarum*, *Bifidobacterium bifidum*, *Bacillus subtilis* fermentation extract, and *Aspergillus niger* fermentation extract) in 45-day-old New Zealand White rabbits for 6 weeks, reported a positive effect on FCR and a higher digestibility of crude protein. Probiotics and digestive enzymes (amylase, cellulase, beta-glucanase, and hemicellulose) present in the added product might also have worked synergistically, and as a result, the gut’s health and environment improved, supporting an improvement in nitrogen utilisation and positively affecting growth. Indeed, the authors also reported an increased weight gain in the final part of the trial in the rabbits fed with higher concentrations of probiotics [24]. Similarly, Lam Phuoc and Jamikorn [26] highlighted that the addition of *B. subtilis* and *L. acidophilus* complex (at, respectively, 0.5 × 10^6^ CFU/g feed and 0.5 × 10^7^ CFU/g feed) increased the FCR in 28-day-old rabbits fed for two weeks (42 days old). This positive effect was then lost, and the results were no longer different from the control diet (tested period 42 days to 70, and also total period 28 days to 70 days old). Then, effects of probiotics could be also related to a specific time of the rabbit’s life and their positive effects could be ascribed only in relation to the physiological status of the animal. Indeed, the FCR data were only partially in line with those of the digestibility trial (took place at day 63 for 5 days), which highlighted better digestibility coefficients of dry matter, organic matter, crude protein, neutral detergent fibre, and gross energy for the rabbits fed the mix of the probiotics and also the diet with only one probiotic strain (*L. acidophilus* at 1 × 10^7^ CFU/g feed). Nonetheless, FCR data also highlighted a synergistic effect of the microorganisms, as the single use of *B. subtilis* (at 1 × 10^6^ CFU/g feed) or *L. acidophilus* did not show the same results as their mix [26].

On the contrary, it must be taken also into consideration the possibility of an antagonistic effect of the microorganisms employed. About this eventuality, El-Badawi et al. [40] reported a negative association between *Bacillus subtilis* and *Saccharomyces cerevisiae*. The authors tested the bacteria alone (0.1% of bacterial dry media of 3 × 10^7^ CFU/g, Enviva PRO, Dupont, USA) and the yeast (0.1% of dry live yeast of 10^8^ CFU/g, RUMI YEAST Sc47, Neovia, France) along with their mix (0.05%, respectively) in comparison to a control diet in 8-week-old New Zealand White rabbits for 10 weeks. The results of the FCR highlighted that the rabbits fed the mix of microorganisms did not differ from the control, whereas the employment of the two probiotics alone increased it. Similarly, digestion coefficients of most measured nutrients (dry matter, organic matter, crude protein, and nitrogen-free extract) were higher in rabbits fed the two diets with the single cell type than their mix and the control (mix similar to control) [40].

Despite the beneficial effects reported by the abovementioned publications, other authors showed a lack of efficiency of probiotics in modifying the live performance of rabbits treated with these types of microorganisms. Some examples of the lack of effects of yeasts used as probiotics could be found in Emmanuel et al. [39], Tag El Din [29], and Rotolo et al. [43]. In the first study, the authors fed rabbits (16-week-old New Zealand White rabbit, 12-week trial) with *Saccharomyces cerevisiae* (Agro-Chemical Company, Nsukka, Nigeria) at the concentration of 0.12 g per kg, highlighting a lack of variations in FCR, final body weight, and average daily weight gain [39]. Similarly, Tag El Din reported that 6-week-old Californian x New Zealand White rabbits fed 0.5, 1.0, and 2.0% dry live yeast inclusion (10 CFU/g, RUMI YEAST Sc 47, Neovia, France) for 5 weeks did not show any statistical differences to the control in the FCR and digestibility coefficient [29]. Noteworthily, inclusion of 1.5% of dry yeast induced a positive variation in FCR. The authors hypothesised that these results may be due to the reduction of toxins or antimicrobial substances produced by other microorganisms, the competition for adhesion to epithelial cells, the increased resistance to colonisation, the stimulation of the immune system of the host, and the reduction of stress in rabbits. Likewise, Rotolo et al. [43] reported no variations in performance parameters by live yeast addition (37-day-old New Zealand White rabbits fed for 47 days with 300 and 600 mg/kg of *Saccharomyces cerevisiae boulardii*, LSB, LEVUCELL^®^ SB10 ME TITAN, Lallemand Sas, Blagnac, France). A lack of effects on live performance (final weight and FCR) was also reported for the employment of some bacteria, as reported by Fathi et al. [25], which tested two supplementation levels (200 or 400 g of probiotic/t feed) of *Bacillus subtilis* (4 × 10^9^ CFU/g) in 8-week-old Jabali breed rabbits (Egyptian local breed) reared at 35 °C for 8 weeks.

### 3.2. Health Status and Gastrointestinal Tract Microbiota

Several authors reported that during the experiments all animals remained in good health condition, with no symptoms of disorders; therefore, no mortality and morbidity were noted [24,28,30,31,32,43]. As reported in the paragraph above, Cunha et al. [34] interrupted the administration of *E. coli* strains isolated from rabbit faeces after morbidity signals such as diarrhoeic faeces with a decrease in feed intake associated with an increase in water consumption. Dimova et al. [42] recorded a lower mortality rate (16.67%) in fattening rabbits (White New Zealand, 14 days old to 101 days old) fed 0.5% of probiotics (Zoovit, no details given) than animals fed the control diet (27.78%). A similar reduction of mortality (−10.8%) was also highlighted in weaned rabbits derived from does fed the probiotic. Similarly, Kimsé et al. [44] reported that growing rabbits (INRA hybrid line, UMR 1289 INRA TANDEM, from day 35 to day 70) fed a supplementation of *S. cerevisiae* strain NCYC Sc 47 (Actisaf: *S. cerevisiae* NCYC Sc 47 coated with saccharides, Lesaffre Feed Additives, Marquette-Lez-Lille, France; at 10 g/kg of basal diet) significantly decreased mortality (22.5%) compared to the control (45.0%). These improvements of digestive health might be associated with the possibility of the probiotic strains to colonise the caecum and then modify the caecal physico-chemical characteristics, such as an increase in redox potential [44].

To evaluate the effect of probiotic supplementation in diets on the immune response, some authors took into account specific and non-specific immune response. For this reason, different haematological parameters were analysed, mainly total protein, immunoglobulins, white blood count (WBC), and lymphocytes. The studies reported variations in the results, and the reasons for that could be related to the use of different types and doses of probiotics, as well as differences in feed composition and mechanism of action which characterised different probiotics.

El-Shafei et al. [37], who tested supplementation of probiotic *Lactobacillus plantarum* in growing New Zealand White rabbits, reported no significant effect of probiotics on total protein, immunoglobulins, and on IgG. The results reported by Mohamed et al. [27] partially agreed with the abovementioned, as no significant effect was reported on globulin, while a significant increase in WBC and total protein in growing rabbits fed with diets supplemented with *Lactobacillus acidophilus* were observed. An increase of total protein along with an increase in globulins was observed by other authors [25,41]. Fathi et al. [25], in local breed growing rabbits fed with two different doses of probiotic containing *Bacillus subtilis*, reported a slight increase of total protein level and a similar trend in globulins with the highest dose of probiotic. Similarly, Abdel-Azeem et al. [41], who employed a commercial probiotic product, obtained higher levels of these parameters in treated groups than the control.

An opposite trend was reported by other authors [33,36]. Beshara et al. [36] reported a decrease of total protein, globulin, and WBC in treated groups with probiotics; likewise, Wlazło et al. [33], who studied the effect of fermented rapeseed meal in rabbit diets on immune status, determining the class A, G, and M immunoglobulins in blood plasma, observed a lower level of class G immunoglobulins in the treated group than the control and a decrease of the IgG values as the level of fermented rapeseed meal increased. The same author also observed a correlation between the IgG values and the numbers of microorganisms in the GIT.

As reported above, the different results obtained probably may be related to different mechanisms of action of probiotics. In fact, the probiotic might be able to (i) modulate the host’s defences, increasing the immunity response or (ii) can exercise a direct effect on microorganisms, e.g., pathogens, reducing the host requirement of immune defences.

Some authors analysed the effects of probiotics on the intestinal morphology. Shen et al. [35] reported that oral administration with *Lactobacillus casei* significantly increased the length of the vermiform appendix in suckling rabbits, but did not change the intestinal morphological indices, including villus height, crypt depth, and the ratio of villus height to crypt depth. As development of the vermiform appendix is associated with the immune capacity of the intestine of a rabbit, the results of this study indicated that the development of the special intestinal immune organ in rabbits needs bacterial stimulation. Moreover, Shen et al. [35] reported an increase in the percentage of degranulated Paneth cells in the duodenum and jejunum of suckling rabbits orally administered with *Lactobacillus casei* RABX1. Degranulation is the way that a Paneth cell secretes its synthesised antimicrobial substances to the intestinal lumen, such as defensin and lysozyme. The increased percentage of degranulated Paneth cells and the expression of toll-like receptors (the first barrier in host defence against pathogen infection that induces production of type I interferons and inflammatory cytokines) in the duodenum and jejunum indicated that the probiotic was involved in the regulation of Paneth cell function in the rabbits.

Liu et al. [28] tested three doses of a probiotic strain of *Clostridium butyricum* (CCTCC AB: 2017089; low dose, 1.0 × 10^3^ CFU/g; medium dose, 1.0 × 10^4^ CFU/g; high dose, 1.0 × 10^5^ CFU/g) in 5-month-old primiparous female rabbits (Sichuan white rex rabbit) until new-born weaning (35 days), and then also fed the weaning rabbits themselves with the same diet of the mothers (4-week feeding trial). The authors reported that compared to the control, rabbits supplemented with a high dose of probiotic elongated the length of the villi of small intestinal tissues, while the medium dose group showed longer villi in the duodenum and ileum. On the other hand, probiotic treatments decreased the crypt depth of weaning rex rabbits. Therefore, the ratios of villus length to crypt depth (VL/CD) were greater in the high dose group than in the control and low dose group. Probiotics can increase villus length and decrease crypt depth in the small intestine, which is beneficial for the digestion and absorption of nutrients, thus directly affecting mucosa morphology, digestive enzyme activity, and consequently growth performance.

El-Shafei et al. [37] tested, in four-week-old male New Zealand White rabbits, two concentrations of a *Lactobacillus plantarum* strain (0.25 g and 0.5 g per kg of 1 × 10^6^ CFU/g) for 8 weeks. Results showed that the goblet cells appeared in the duodenum and caecum epithelia of the treated groups, suggesting an improvement in production of mucus compared with the control group. Meanwhile, the group fed 0.5 g probiotic/kg diet showed improvement in goblet cells and crypts in the base of the tissue or surface compared to the control group. These results confirmed the increased health status of treated rabbits as the enhanced mucus layer covering the epithelial lining of the gut can serve as an antibacterial shield that prevents the binding of enteric pathogens, and goblet cells have a role in defence at the intestinal mucosa.

Not all the studies about the dietary administration of probiotics in rabbits reported modification in the intestinal morphology. For instance, Pogány Simonová et al. [32] and Oso et al. [45] did not report modification in jejunal morphometry or morphological parameters in the rabbit ileum after probiotic inclusion (*E. faecium* and a mix of *Pediococcus acidilactici* and *Bacillus cereus*, respectively).

All the research studies that evaluate GIT microbiota found modification in microorganisms’ populations of GIT in relation to probiotic addition. Wlazło et al. [33] tested the effects of the administration of a fermented rapeseed meal with *Bacillus subtilis* as the probiotic (strain 87Y from the collection of InventionBio Ltd., Bydgoszcz, Poland) in 35-day-old New Zealand White × Popielno White rabbits for 85 days. The authors enumerate few microbial species in the duodenum, small intestine, caecum, and colon sections. Duodenum, small intestine, and colon lactic acid bacteria were increased due to probiotic addition, as well as small intestine mesophilic aerobic bacteria. No variation was detected in number of total fungi in all the sections. Noteworthily, a number of coliforms and *Escherichia coli* decreased in the small intestine and colon sections in relation to the probiotic diets [33]. A *Bacillus subtilis* strain, alone and in association with *Lactobacillus acidophilus* strain (also tested alone), were also tested by Lam Phuoc and Jamikorn [26]. The addition of the *Bacillus subtilis* probiotic strain (alone and in association with *Lactobacillus acidophilus* probiotic strain) increased the numbers of bacilli in the ileum and colon, and generally, an increment of the numbers of bacilli were observed in all segments in the rabbits supplemented with either one of the probiotics. Similarly, the average number of lactobacilli in all intestinal segments of the rabbits were increased after the probiotic diets. The authors hypothesise a synergistic effect between *B. subtilis* and *L. acidophilus*. On the other hand, no difference was observed in the ileum coliform number, even if *L. acidophilus* showed an effect on coliform numbers in the cecum and colon, and an average number in all segments. These variations led, in rabbits fed *L. acidophilus* probiotics, to an increase in Gram-positive bacteria (lactobacilli) and a reduction in Gram-negative bacteria (coliforms).

An increment in lactobacilli, due to probiotic diets, was also reported by Shen et al. [35] and Abdel-Azeem et al. [41]. New Zealand White rabbits were fed *Lactobacillus casei* RABX1 (accession number: KT944253) resuspended in the milk (5–6 × 10^8^ CFU/mL) and orally administered from 5 to 13 days of age. The probiotic milk feeding was suspended two days before slaughtering (slaughtered at 15 days old) [35]. Shen et al. reported that *Lactobacillus casei* significantly increased the length of the vermiform appendix in suckling rabbits without modifying the intestinal morphological indices, villus height, crypt depth, and the ratio of villus height to crypt depth. The content of the small intestine showed to be affected by the diet; suckling rabbits fed the “probiotic milk” presented a higher relative proportion of *Lactobacilli* in total intestinal bacteria and a lower relative proportion of *Escherichia*–*Shigella* than rabbits fed the control milk. Abdel-Azeem et al. [41] employed a commercial probiotic product (ZAD^®^, Academy of Scientific Research and Technology, Egypt) in an oral gavage administration in 6-week-old New Zealand White rabbits for 56 days. The probiotic product (mix of enzymes and bacteria) increased the concentration of *Lactobacillus* spp. in the caecum and decreased the total coliform and total anaerobic bacteria. On the other hand, Beshara et al. [36] reported a lack a variation in lactic acid bacteria in rabbits fed probiotics, whereas an increase of the total bacterial count was detected.

Some research studies were also carried out on probiotic strains isolated from rabbits itself. Cunha et al. [34] tested, in 38-day-old New Zealand White rabbits, the administration of enterococci and three *E. coli* strains isolated from rabbit faeces. Interestingly, the administrations of *E. coli* were interrupted as the animals showed, between the second and fifth day of the trial, diarrhoeic faeces and gastrointestinal signs with a decrease in feed intake and an increase in water consumption. On the other hand, *Enterococcus* spp. showed a positive interaction with the hosts even if the authors highlighted those probiotic bacterial strains did not remain in the gastrointestinal tract longer than one week after the administration ended. Cunha et al. hypothesised that this might be caused by an impaired probiotic persistence in the rabbits’ intestinal microbiota due to a lack of (re)inoculation, a dietary change, a different water source, and/or a new household.

The use of *Enterococcus* spp. was also taken into account by Pogány Simonová et al. [32] using a bacteriocin-producing strain (*Enterococcus faecium* EF9a) with probiotic properties isolated from the faeces of the Hungarian Pannon White rabbit breed [46]. The probiotic strain *Enterococcus faecium* EF9a was added to the drinking water and induced a significant decrease in the coliforms, coagulase-positive staphylococci, pseudomonads, and coagulase-negative staphylococci in the rabbit faeces, as well as showing antimicrobial effects in the caecum against coliforms, coagulase-negative staphylococci, and pseudomonads and in the appendix versus the coliforms [32]. Furthermore, Lauková et al. [30] tested an *Enterococcus faecium* strain. The employed strain, an environmental isolate, was also an enterocin M producer (strain AL41, registered in Czech Culture Collection, Brno, Czech Republic—CCM8558), which was tested alone and in association with *Eleutherococcus senticosus* (Siberian ginseng), a herb with adaptogenic, anti-stress, and immunomodulatory properties. The authors reported that *E. faecium* AL41 colonised the rabbits’ GIT better when employed alone than in association with the herb, and it remained present in the animals after 3 weeks of its cessation. Significant reductions of coagulase-negative staphylococci, coagulase-positive staphylococci, *Clostridia*, coliforms, and/or pseudomonads were also highlighted in relation to *E. faecium* AL41 administration. Changes in microbiota were associated both to the antibacterial effect of the produced enterocin M as well as to the production of lactic acid from the probiotic strain.

### 3.3. Carcass and Meat Quality

As reported for the live performance, carcass traits and meat quality were also partially affected by probiotic supplementation.

Some authors reported that probiotics ameliorate the carcass traits, even increasing the edible parts. Fathi et al. [25] showed that rabbits fed *Bacillus subtilis* supplementation (200 and 400 g/t) increased the carcass weight (only 400 g/t), dressing percentage, and cuts of mid part and hind part as a percentage of live body weight. Interestingly, the authors reported that rabbits fed 400 g/t of *Bacillus subtilis* at 4 × 10^9^ CFU/g for 8 weeks increased the carcass weight, on average by 130 g, corresponding to an increase of 12% of the weight of the carcasses derived from the rabbits fed the control diet. These data are even more outstanding if we take into consideration that the final live weight was not statistically different between the two diet groups and that rabbits were reared under a hot climate (35 °C). Likewise, Mohamed et al. [27] reported that most of the carcass traits studied were affected in rabbits fed probiotics compared with the control group (two breeds, New Zealand White and local Egyptian breed called Baladi Black; five different probiotic diets, dose per day: 1 mL fresh culture suspension of *Bifidobacterium bifidum* 1 × 10^7^ CFU, 1 mL fresh culture suspension of *Lactobacillus acidophilus* 7 × 10^6^ CFU, 1 mL fresh culture suspension of bacterial mixture of *Bifidobacterium bifidum* and *Lactobacillus acidophilus* at 3.5 × 10^7^ CFU, and 1 mL of *Saccharomyces cerevisiae*). The authors also highlighted that the local breed, Baladi Black, fed *Bifidobacterium bifidum* supplementation recorded the highest values of carcass weight, carcass percentage, and heart, liver, kidneys, lungs, and giblets (absolute weights) in comparison with the other groups.

Carcass traits were not affected in other research trials, such as the data reported by Beshara et al. [36], who tested 0.4 g/kg of a thermo stable probiotic (*Lactobacillus lactis* 2.5 × 10^8^ CFU/kg, *Bacillus subtilis* 1.8 × 10^9^ CFU/kg—calcium carbonate up to 1 g as carrier, Saltose Ex, Pic-Bio Inc. Company, Shinagawa, Japan) in the same local Egyptian breed (Baladi Black) employed by the former authors. Moreover, the employment of bacteria or yeast alone or in combination did not affect the carcass traits also, as reported by El-Badawi et al. [40] in New Zealand White rabbits. No differences in carcass characteristics among treatments were also reported in Rotolo et al. [43].

Interestingly, Abdel-Wareth et al. [24] reported even a negative effect on the carcass yield percentage in relation to the probiotic administration. Noteworthily, it is important to highlight that Abdel-Wareth et al. tested three different diets containing increasing concentrations of probiotics in relation to increasing dietary dry fenugreek seeds. It is reported that some cultivars of fenugreek (*Trigonella foenum-graecum*) could also bring some antinutritional factors, such as phytic acid, also present in its seeds [47]. Abdel-Wareth et al. also reported that rabbits fed the highest concentration of dry fenugreek seeds/probiotics (diet with 15 g/kg dry fenugreek seeds and 450 mg/kg probiotic—AmPhi-Bact^®^) showed lower caeca weight (percentage of slaughter weight) than the control diet. On the contrary, Rotolo et al. [43] reported that caecum weight was not affected by treatment in weaning rabbits upon dietary inclusion of a probiotic (live *Saccharomyces cerevisiae boulardii*).

Meat quality of rabbits fed probiotics showed as well mixed results. No variations in the pH_48_, colour, proximate composition, and water holding capacity was reported by Pogány Simonová et al. [31], who tested in 5-week-old Hyplus breed rabbits for 28 days a strain of *Enterococcus faecium* (EF9a isolated from Pannon White rabbit, 1 × 10^9^ CFU/mL, in a dose 500 µL/animal/day) in the drinking water. Moreover, El-Badawi et al. [40] and Islamov et al. [38] did not find, respectively, significant differences in rabbit meat quality (proximate composition) with the use of alone or combined bacteria yeast supplements (*Bacillus subtilis* and *Saccharomyces cerevisiae*) and a probiotic preparation called “Rescue Kit” (1 kg of preparation contains 800 × 10^9^ CFU *Bacillus subtilis* and 800 × 10^9^ *Bacillus licheniformis*; inclusion level of 10 g of preparation per 1 kg of feed; White Giant breed; from 70 days old to 120). No significant effects of live *Saccharomyces cerevisiae boulardii* supplementation were observed also by Rotolo et al. [43] in pH_24_, colour, cooking loss, and proximate composition of *longissimus dorsi* muscle.

Fathi et al. [25] reported instead modifications of the proximate composition of rabbits fed *Bacillus subtilis* as a probiotic. The authors reported higher percentages of dry matter, organic matter, protein, and fat in rabbits fed a diet containing 400 g/t probiotics than the control one and probiotics at 200 g/t. As well, Abdel-Wareth et al. [24] reported a decrease in water holding capacity (estimated by centrifuging the muscle) and cooking loss in a group of rabbits fed combinations of fenugreek seeds and probiotics (diet with 5 g/kg dry fenugreek seeds and 150 mg probiotic—AmPhi-Bact^®^). As reported by Pogány Simonová et al. [31], no variation was detected in meat pH values (in this case measured 24 h post-mortem).

## 4. Conclusions

Some evidence suggests that probiotics can play several roles in rabbit farming, from biological control against pathogenic microorganisms, to growth enhancer or active compounds to increase the quantity and quality of the final product. The positive effects determined in rabbits fed the experimental diets are in general ascribed to the capacity of probiotics to interact with the host and with all the microbiota present in the different parts or organs of the GIT, modifying the entire production process. From the analysis of the different research studies emerges the findings that probiotics have different modes of action and that many factors can modify the responses, confirming that probiotic properties have bacterium–host specificity. Research of this type is especially important in terms of reducing the use of antibiotics for therapeutic purposes through nutritional prevention in animals.

Attention must be taken to the correct time of administration and also in relation to the probiotic activities and amounts. Optimisation of the microbiota composition leads to an increase in digestive efficiency, directly improving nutrient digestibility and stimulating immune processes, increasing the profitability of production.

Future research must be focused also on the technologies of the administration (microencapsulation, cell immobilisation, and continuous fermentation) to ensure that these beneficial microorganisms will reach, in high numbers, the target site of action.

## Figures and Tables

**Table 1:** Experimental designs of the reviewed articles.

Ref.	Probiotic	Probiotic Diets	Breed	Total Animals	Start Age, Days	Diet Length, Days	Climate
[24]	AmPhi-Bact ^a^	-5 g/kg of dry fenugreek seeds and 150 mg/kg probiotic -10 g/kg dry fenugreek seeds and 300 mg/kg probiotic -15 g/kg dry fenugreek seeds and 450 mg/kg probiotic	NZW	128	45	42	22 °C
[25]	*Bacillus subtilis*	-200 g of probiotic */t feed -400 g of probiotic */t feed * Bacillus subtilis 4 × 10^9^ CFU/g	Jabali Spanish breed (V-Line) crossbreds ¼J¾V crossbreds ¾J¼V	20 20 20 20	56	56	20–35 °C
[26]	*Bacillus subtilis* *Lactobacillus acidophilus*	*-Bacillus subtilis* 1 × 10^6^ CFU/g feed *-Lactobacillus acidophilus* 1 × 10^7^ CFU/g feed *-Bacillus subtilis* 0.5 × 10^6^ CFU/g feed + *Lactobacillus acidophilus* 0.5 × 10^7^ CFU/g feed	NZW	64	28	42	26.6–33.8 °C
[27]	*Bifidobacterium bifidum* *Lactobacillus acidophilus* *Saccharomyces cerevisiae*	-1 mL of *Bifidobacterium bifidum* 1 × 10^7^ CFU/day/rabbit -1 mL of *Lactobacillus acidophilus* 7 × 10^6^ CFU/day/rabbit -1 mL of *Bifidobacterium bifidum* + *Lactobacillus acidophilus* 3.5 × 10^7^ CFU/day/rabbit -1 mL of *Saccharomyces cerevisiae*/day/rabbit (CFU not reported)	NZW Baladi Black	75 75	35	56	20–27 °C
[28]	*Clostridium butyricum* CCTCC AB: 2017089	-1.0 × 10^3^ CFU/g -1.0 × 10^4^ CFU/g -1.0 × 10^5^ CFU/g	Sichuan white rex	60 (120) ^b^	35	28	-
[29]	Dry live yeast (RUMI YEAST-*Saccharomyces cerevisiae* Sc 47-Neovia-France)	-0.5% dry live yeast inclusion of 10 CFU/g -1.0% dry live yeast inclusion of 10 CFU/g -1.5% dry live yeast inclusion of 10 CFU/g -2.0% dry live yeast inclusion of 10 CFU/g	Californian × NZW	60	42	35	-
[30]	*Enterococcus faecium* AL41(Czech Culture Collection of Microorganisms number CCM8558)	*-Eleutherococcus senticosus* (Siberian ginseng) -500 μL *Enterococcus faecium* AL41 10^9^ CFU/mL/animal/day in the water -Siberian ginseng + 500 μL *Enterococcus faecium* AL41 10^9^ CFU/mL/animal/day in the water	Hyplus	96	35	21	16 °C
[31,32]	*Enterococcus faecium* EF9a bacteriocin-producing strain	-Ringer solution with *Enterococcus faecium* EF9a 1.0 × 10^9^ CFU/mL, in a dose 500 µL/animal/day into drinking water	Hyplus	48	35	28 (42) ^c^	16 ± 4 °C
[33]	Fermented rapeseed meal with *Bacillus subtilis* (87Y strain collection of InventionBio Ltd., Bydgoszcz, Poland)	-4% of fermented rapeseed meal -8% of fermented rapeseed meal -12% of fermented rapeseed meal	NZW × Popielno White	40	35	85	climate-controlled building
[34]	Four strains of *Enterococcus* spp. Three strains of *Escherichia coli*	-1 mL/kg *Enterococcus* spp. at 5.0 log CFU/mL every 72 h -1 mL/kg *Escherichia coli* at 5.0 log CFU/mL every 72 h	NZW	12	38	25	22 °C
[35]	*Lactobacillus casei* RABX1 (accession number: KT944253)	-Resuspended in the milk (5–6 × 10^8^ CFU/mL) and orally administered	NZW	24	5	8 (10) ^d^	30 °C
[36]	*Lactobacillus lactis* *Bacillus subtilis*	-0.4 g/kg of *Lactobacillus lactis* 2.5 × 10^8^ CFU/g + *Bacillus subtilis* 1.8 × 10^9^ CFU/g ^e^	Black Baladi	54	42	42	-
[37]	*Lactobacillus plantarum*	-0.25 g of probiotic */kg -0.5 g of probiotic */kg * *Lactobacillus plantarum* 1 × 10^6^ CFU/g	NZW	36	28	56	Egyptian environmental conditions (December–January)
[38]	Rescue Kit ^f^	-10 g Rescue Kit/kg feed	White Giant	28	70	50	-
[39]	*Saccharomyces cerevisiae*	-0.12 g/kg *Saccharomyces cerevisiae* -150 mg/kg zinc oxide -0.12 g/kg *Saccharomyces cerevisiae* + 150 mg/kg zinc oxide	NZW	16	112	84	-
[40]	*Saccharomyces cerevisiae* *Bacillus subtilis* ^g^	-0.1% *Bacillus subtilis* * -0.1% *Saccharomyces cerevisiae* ** -0.05% *Bacillus subtilis* * + 0.05% *Saccharomyces cerevisiae* ** * *Bacillus subtilis* 3 × 10^7^ CFU/g ** *Saccharomyces cerevisiae* 10^8^ CFU/g	NZW	60	56	70	-
[41]	ZAD ^h^	oral gavage -0.25 mL/rabbit/day -0.5 mL/rabbit/day -1.0 mL/rabbit/day	NZW	180	42	56	-
[42]	ZOOVIT ^i^	-0.5% probiotic	NZW	36 ^j^	14	87	-

NZW: New Zealand White. ^a^ American Pharmaceutical Innovations Company^®^, containing a mix of lactic acid bacteria culture, *Lactobacillus acidophilus*, *Lactobacillus plantarum*, *Bifidobacterium bifidum*, *Bacillus subtilis* fermentation extract, and *Aspergillus niger* fermentation extract. ^b^ Sixty 5-month-old healthy primiparous females were fed the diets, then the weaning rabbits followed the diets of the mother for 4 weeks. ^c^ Probiotic administered for 28 days, sampling continued for 14 days. ^d^ Slaughtered at 15 days old after 2 days of suspension. ^e^ Tested also in relation to feed restriction. ^f^ Probiotic preparation with 800 × 10^9^ CFU/kg of *Bacillus subtilis* + 800 × 10^9^ CFU/kg of *Bacillus Licheniformis*. ^g^ Dry live *Saccharomyces cerevisiae* Sc47 (RUMI YEAST, Neovia, France) and bacterial dry media of *Bacillus subtilis* (Enviva PRO, Dupont, USA). ^h^ Patented product manufactured by the Academy of Scientific Research and Technology, Egypt. ^i^ Products containing mainly bacteria of the genera Lactobacilli, Bifidobacteria, *Lactococcus*, *Bacillus,* and *Pediococcus*, and yeasts (*Saccharomyces* strains). ^j^ 50 does (diet length not reported), 36 fattening rabbits.
